# Copper nanocubes as low-cost enzyme mimics in a sarcosine-sensing reaction cascade†

**DOI:** 10.1039/d4an01242a

**Published:** 2025-02-11

**Authors:** Anuja Tripathi, Mark P. Styczynski

**Affiliations:** a School of Chemical and Biomolecular engineering, Georgia Institute of Technology 950 Atlantic Dr Atlanta Georgia 30332 USA atripathi84@gatech.edu mark.styczynski@chbe.gatech.edu

## Abstract

The development of simple, inexpensive, deployable clinical diagnostics could have a global impact on public health by making measurements of patient health status more widely accessible to patients regardless of socioeconomic status. Here, we report a novel biosensor for sarcosine using a colorimetric readout created by a hybrid catalyst system using copper nanocubes and the enzyme sarcosine oxidase. The enzyme catalyzes the reaction of sarcosine to generate H_2_O_2_, which the copper nanocubes then use as a substrate to create free radicals that convert colorless 3,3′,5,5′-tetramethylbenzidine (TMB) to its blue, oxidized form. The sensor showed good substrate affinity for Cu nanocubes and yielded a wide linear response range (0–140 μM) for sarcosine detection, with high selectivity against various interfering species. The limit of detection and limit of quantification were found to be 1.43 μM and 4.7 μM, respectively. We showed that the biosensor maintains function in a complex serum sample matrix, suggesting potential utility in clinical applications. Finally, we demonstrated a prototype based on light emitting diodes (LEDs) and a light-dependent resistor (LDR) for unambiguous visual interpretation using an inexpensive microcontroller potentially suitable for use outside of traditional clinical or analytical laboratories.

## Abstract 2

Copper nanocubes as low-cost enzyme mimics for sarcosine sensing.

## Graphical abstract

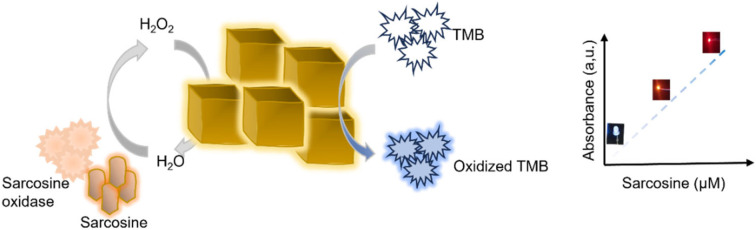

## Introduction

Sarcosine (*N*-methylglycine), a non-proteinogenic amino acid produced in the human body during glycine metabolism, has been identified as a valuable clinical biomarker for a variety of conditions.^1^ The reference ranges of sarcosine in serum is 1.4 ± 0.6 μM.^2^ Elevated sarcosine levels have been associated with diseases including prostate cancer, HIV infection, and cardiovascular diseases, which affect patients across the socioeconomic spectrum.^3–5^ Early identification of such diseases generally offers the opportunity for more successful patient outcomes when the conditions might be more easily treated.^6^

While measuring sarcosine is reasonable in regions with well-established healthcare infrastructure, it is not feasible for global use in areas with limited resources. Currently, various complex methods including capillary electrophoresis, high-performance liquid chromatography, and electrochemical approaches are used for sarcosine quantification.^7^ However, these methods have limitations that prevent them from having a broader impact on public health, such as reliance on expensive solvents, use of expensive equipment, and intricate sample processing requirements. Thus, the development of easy-to-use, inexpensive, and deployable analytical methods to detect sarcosine in biological fluids would have a significant impact. In contrast, colorimetric detection methods are easy to perform with minimal training, are low-cost, and provide easily interpretable output signals without complex equipment. These characteristics make colorimetric methods an attractive option for deployable and widely accessible measurement of various biomarkers, including sarcosine.^8^

**Scheme 1:**
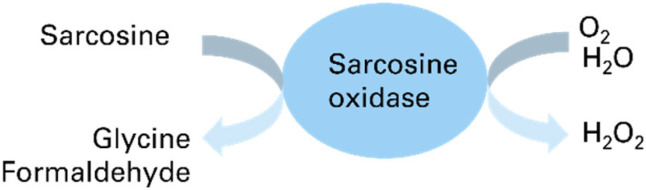
Reaction scheme for sarcosine catalyzed by sarcosine oxidase enzyme.

One widely used approach for measuring metabolites in research settings entails the use of enzymes to catalyze a sequence of chemical reactions leading to the production of a colorimetric output. The first enzyme in the sequence often provides specificity for the target molecule of interest, and subsequent reactions are performed to transform the chemical signals created by the first enzyme into visual output. In the case of sarcosine, the enzyme sarcosine oxidase (Sox) catalyzes the formation of hydrogen peroxide *via*Scheme 1:

Hydrogen peroxide can in turn be used by the enzyme horseradish peroxidase (HRP), which can oxidize the colorless molecule 3,3′,5,5′ tetramethylbenzidine (TMB) to a blue oxidized form (oxTMB).^11^ The intensity of this color, which can be read *via* absorbance at 652 nm, is directly proportional to the concentration of sarcosine in the solution. However, the use of multiple enzymes in an assay has multiple drawbacks, including the cost of purifying enzymes and their different optimal pH, temperature, and chemical environments that can lead to degraded function or even denaturation during storage or reaction.^9^ It is thus desirable to, when possible, replace enzymes with more robust and cost-effective substitutes.

One promising class of substitutes for enzymes is nanozymes, which are nanomaterials that mimic enzymatic catalytic activities.^9–14^ Nanozymes have been shown to have distinct advantages over natural enzymes, including their potential for operation or storage under harsh temperature and pH, and have been used for various applications including biocatalysis and biosensors.^10,11,15–17^ One reason that nanozymes can efficiently mimic enzymes is that they are typically made from the metals that natural enzymes often rely on as active centers for their catalytic functions, particularly iron (Fe), manganese (Mn), copper (Cu), or zinc (Zn). For instance, copper serves as the active center for various proteins and enzymes including laccase, copper–zinc superoxide dismutase, cytochrome oxidase, tyrosinase, and others.^18–21^ These copper-containing enzymes play roles in electron transfer, redox reactions, oxygen molecule transport, and organismal activation.

Peroxidase activity has been previously shown to be a functionality that can be mimicked by nanozymes.^22–28^ A wide range of nanozymes like metal–organic frameworks, metals, metal oxides, quantum dots, and carbon–based nanomaterials^11,29^ have been explored for this purpose since the first magnetic Fe_3_O_4_ nanoparticles were discovered to have peroxidase-like properties in 2007.^30^ However, noble metals that are often used in these nanozymes have high cost and limited availability, while transition nanomaterials in 2D or 3D forms like graphene,^31^ hexagonal boron nitride,^32^ and g-C_3_N_4_^33^ typically either require tedious fabrication techniques or cannot be reused. Copper is an inexpensive substrate with significant potential to mimic enzymatic functionality. To date, only a few copper-based nanomaterials with peroxidase-like activities have been studied, including copper oxide nanoparticles, copper sulfide nanomaterials, protein-copper sulfate nanoflowers, and copper hydroxide nanocages.^34,35^ However, these materials also involve complex fabrication techniques, and they are typically solution-phase, meaning that it would be challenging to implement them in a reusable sensing device.

In this work, we have created nanozymes to mimic peroxidase-like properties using Cu nanocubes formed *via* simple electrochemical etching of a plain, inexpensive copper substrate. These nanozymes were used to replace the horseradish peroxidase activity in a sarcosine assay by catalyzing the reaction of hydrogen peroxide with TMB to produce a visible blue color. We demonstrate that since these Cu nanocubes are anchored on a surface, they can easily be recovered and reused. We established optimized reaction conditions for nanocubes synthesis and for the TMB oxidation reaction in the presence of Cu nanocubes. We compared the catalytic activity of Cu nanocubes with a plain Cu substrate. Combining the Cu nanocubes with Sox enzyme, we investigated the system's ability to detect sarcosine under physiologically relevant conditions, including assessment of selectivity, limit of detection, and reusability. Finally, we demonstrated a proof-of-concept device on a breadboard for unambiguous color-based semi-quantitative detection *via* an LED readout.

## Results and discussion

### Fabrication and characterization of Cu nanocubes

**Fig. 1:**
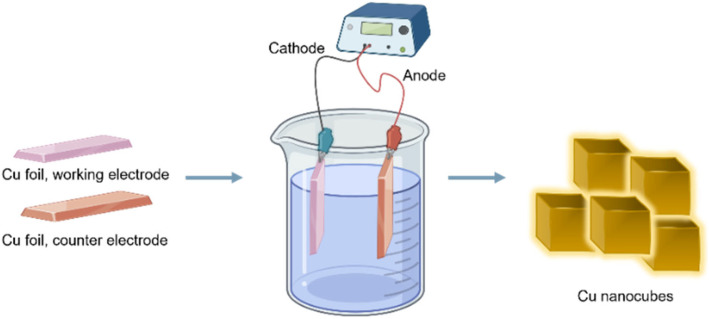
Electrochemical synthesis of Cu nanocubes at 8V in 1 M CuCl_2_ electrolyte.

**Fig. 2:**
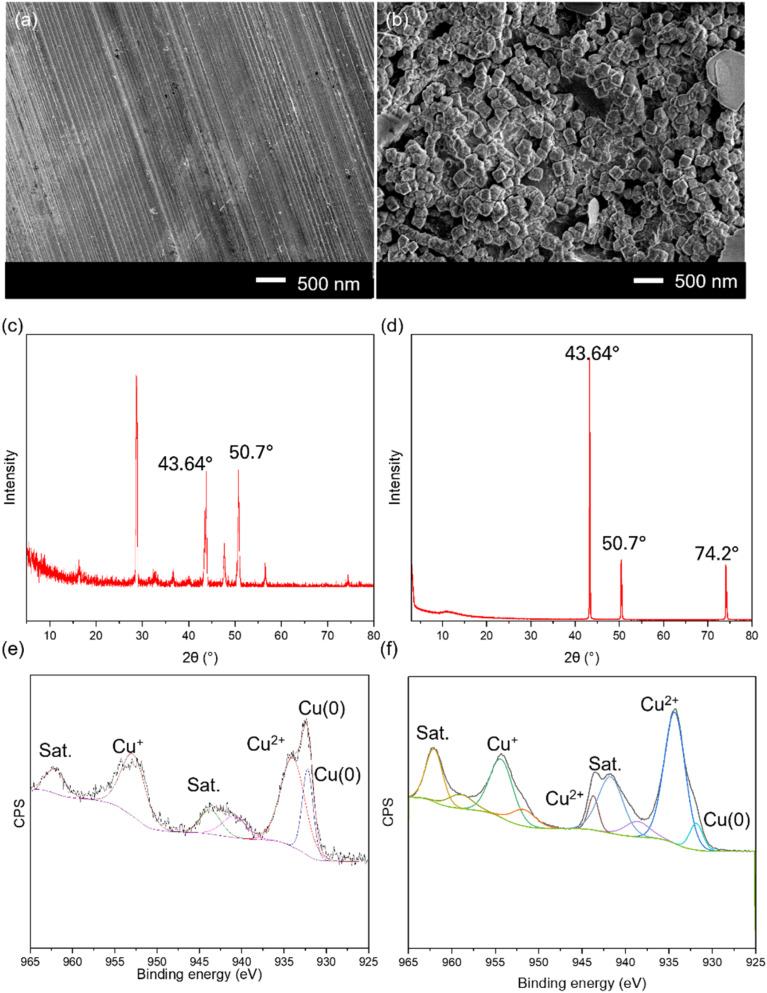
Characterization of copper nanocubes synthesis, including SEM images of pristine Cu foil (a) and Cu nanocubes (b), XRD spectra of Cu pristine Cu foil (c) and Cu nanocubes (d), and XPS spectra of pristine Cu foil (e) and Cu nanocubes (f). XPS experimental spectra are represented in black. The spectra were deconvoluted into their individual component peaks. Cu nanocubes were fabricated using electrochemical etching for 2 min using a DC power supply at 8 volts and 4 amperes.

Copper nanocubes were synthesized using electrochemical etching in 1 M CuCl_2_ at 8 volts and 4 amperes of current (Fig. 1) across various time intervals from 30 s to 5 min. The electrolyte dissociates through ionization, leading to the reduction of copper ions at the cathode, where copper metal is deposited. Copper is removed from the anode and oxidized to Cu^2+^ ions, as shown in the following reactions:

We observed that as the etching time increased from 30 s to 60 s, the copper foil at the anode began to deform (Fig. S1† and Fig. 2), leading to the formation of defects and structures. Notably, at 2 minutes of etching time, the surface showed a consistent distribution of uniform Cu nanocubes across the entire surface; the pristine copper foil (Fig. 2a) did not exhibit any structure on its surface. Beyond 2 minutes of etching time, the surface morphology began to deteriorate and lose its uniformity, likely due to excess etching, leading to non-uniform dissolution and the nanostructure disappearing.^36^

The crystal phase structure of both pristine Cu and Cu nanocubes was examined through the X-ray diffraction technique (XRD) as shown in Fig. 2c and d. Peaks at 2*θ* values of 43.64° and 50.7° are present, corresponding to (111) and (200) planes of Cu, in comparison with JCPDS # 04-0836.^37^ These peaks are also observed in the diffractogram for the etched Cu foil, with one additional peak attributed to the (220) plane observed at 74.2° (JCPDS # 70-3038).^38–40^ The emergence of this new crystal phase of cubic Cu metal is likely a result of the oxides deposited during electrochemical etching process, but the overall XRD pattern indicates that the entire structure has not fully transitioned to this new phase. In summary, the XRD data suggests that pristine Cu foil displays distinct Cu peaks, while the electrochemically etched Cu foil shows structures that include both Cu and its oxide peaks.

X-ray photoelectron spectroscopy (XPS) analysis revealed the surface chemical composition and elemental valence states of both pristine and electrochemically etched Cu films, as illustrated in Fig. 2e and f. Peaks at 931.9 eV and 932.4 eV signify the presence of metallic Cu for the pristine foil,^41^ while the disappearance of the peak at 932.4 eV for the Cu nanocubes suggests the conversion of metallic Cu to Cu^2+^. Cu^2+^ peaks at 934.4 eV, 934.05 eV, and 944.5 eV (along with satellite (sat.) peaks at 941.84 eV, 943.8 eV, and 941.1 eV) confirm the existence of Cu^2+^ on both pristine Cu and Cu nanocubes, likely in the form of oxides.^42,43^ Peaks at 954.26 eV and 953.1 eV indicate Cu^+^, with its satellite peak at 962.1 eV.^44,45^ This indicates the additional charges on the etched Cu compared to the pristine Cu sample, facilitating fast electron shuttling and catalyzing the TMB-H_2_O_2_ reaction.

### Peroxidase-like activity and steady state kinetic study

We observed that Cu nanocubes were able to mimic peroxidase-like activity by catalyzing the oxidation of TMB, as shown in Fig. S2.† This reaction likely happens through nanozyme interaction with the initial substrate, H_2_O_2_, to produce hydroxyl radicals (˙OH), which then oxidize hydrogen donor molecules such as TMB. This activity and mechanism have been previously reported and characterized for other nanomaterials, including copper-based nanomaterials, by Sielska *et al.*,^46^ Illakkia *et al.*,^47^ and Chen *et al.*^48^ Given limited previous work specifically on etched Cu nanozymes as TMB oxidation catalysts, we first sought to characterize TMB oxidation in the absence of sarcosine and Sox enzyme using absorbance at 652 nm. Reaction temperature, pH, and contact time were varied at a fixed TMB concentration of 1 mM and H_2_O_2_ concentrations of 2 μM, 60 μM, and 60 μM, respectively, for 0.5 cm^2^ of etched copper foil (Fig. S3†). We found that all three variables had a significant impact on TMB oxidation, with local optimum values. The lower measured absorbance at extreme pH and higher temperatures is likely due to the reduced stability of oxTMB (and not necessarily the nanocubes) under these conditions,^49,50^ yielding an apparent decrease in activity. Similarly, the lower measured absorbance after 30 minutes may occur due to the decomposition of oxTMB when left in solution for an extended duration.^51^ The optimal temperature of 40 °C for reaction with the Cu nanocubes was consistent with what one would expect for natural enzymes; for example, the optimal temperature for a reaction with horseradish peroxidase has been previously reported^52^ to be 45 °C. The optimal pH value of 3, however, was quite low; for example, horseradish peroxidase has an optimal pH of 7, with less than 50% activity at a pH of 4 and less than 20% activity at a pH of 9. We chose to operate our nanocube-based sensor at a pH of 5 and a temperature of 37 °C because it would still provide sufficient activity while being more physiologically relevant and enable better compatibility with natural enzymes that might be used in an assay.

**Fig. 3:**
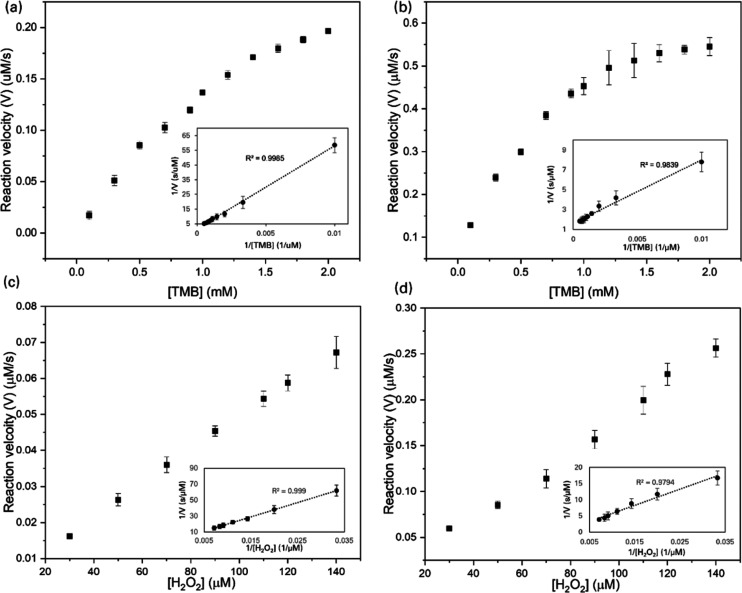
Characterization of reaction kinetics. Initial reaction velocity is plotted as a function of TMB (a and b) and H_2_O_2_ (c and d) substrate concentration for pristine Cu foil (a and c) and etched Cu foil (b and d), with inset Lineweaver–Burk plots showing good fitting to the Michaelis–Menten equation. Standard deviations are from three replicate measurements.

To further characterize the reaction catalyzed by the etched Cu nanozymes, we measured initial reaction kinetics for both pristine Cu and Cu nanocubes at the selected reaction temperature (Fig. 3). The initial reaction velocity curves as a function of TMB concentration resembled those of Michaels–Menten saturation kinetics, so we fit the results to a Michaelis–Menten equation. At low concentrations, the reaction rate increases approximately linearly with TMB and peroxide concentration. Beyond a certain TMB concentration, the initial reaction rate saturates, likely due to the density of catalytically active sites becoming the limiting factor for the reaction. We determined the Michaelis–Menten constant (*K*_m_), representing the affinity of the nanozyme for the substrate and the substrate concentrations where the initial reaction rate is half of its maximum, for both pristine and etched Cu foil. The estimated *K*_m_ values for TMB and H_2_O_2_ were 3.1 mM and 0.93 mM for pristine Cu, and 0.39 mM and 0.65 mM for etched Cu nanocubes. A lower *K*_m_ value is desirable because it indicates that the etched catalyst can act efficiently on lower concentrations of substrate. The kinetic parameters for oxidation of TMB in the presence of H_2_O_2_ by Cu nanocubes compared favorably with values previously obtained for other catalysts (Table 1), with the affinity being among the best previously reported and the maximum reaction velocity being over four times greater than the best previous report. Since the maximum reaction velocity is particularly critical in making a fast, visually interpretable biosensor, these results support the potential utility of the etched copper nanozyme for sensing applications.

**Table 1:** *K*_m_ Values for different peroxidase-mimicking materials

Catalyst	*K*_m_ (mM)		*V*_max_ (M s^−1^)		Ref.
	TMB	H_2_O_2_	TMB	H_2_O_2_	
MoSe_2_ NPs	0.014	0.155	50.56 × 10^−8^	0.99 × 10^−8^	53
Cu/CN	0.04	5.52	6.35 × 10^−8^	12.95 × 10^−8^	54
[Cu (PDA)(DMF)]	0.169	28.6	2.19 × 10^−8^	3.16	55
Co_3_(PO_4_)_2_·8H_2_O	0.136	0.073	0.8 × 10^−8^	1.2 × 10^−8^	56
Ni-Cu_2_O	0.8	1.8	8.6 × 10^−8^	15.2 × 10^−8^	57
Cu–N–C SAzymes	3.76	19.94	75.05 × 10^−8^	20.07 × 10^−8^	58
HRP	0.43	3.7	10 × 10^−8^	8.7 × 10^−8^	30
CS-nFs	237.990	0.068	51.4 × 10^−8^	16.6 × 10^−8^	59
Au/Co_3_O_4_-CeOx NCs	0.1222	0.272	0.8 × 10^−8^	0.4 × 10^−8^	60
6Fe/CeO_2_	0.176	47.6	8.6 × 10^−8^	16.6 × 10^−8^	61
Co_3_(PO_4_)_2_·8H_2_O	0.136	0.073	0.8 × 10^−8^	1.2 × 10^−8^	62
Etched Cu	0.39	0.65	6.17 × 10^−7^	14.2 × 10^−7^	This work

### Determination of H_2_O_2_ and sarcosine

**Fig. 4:**
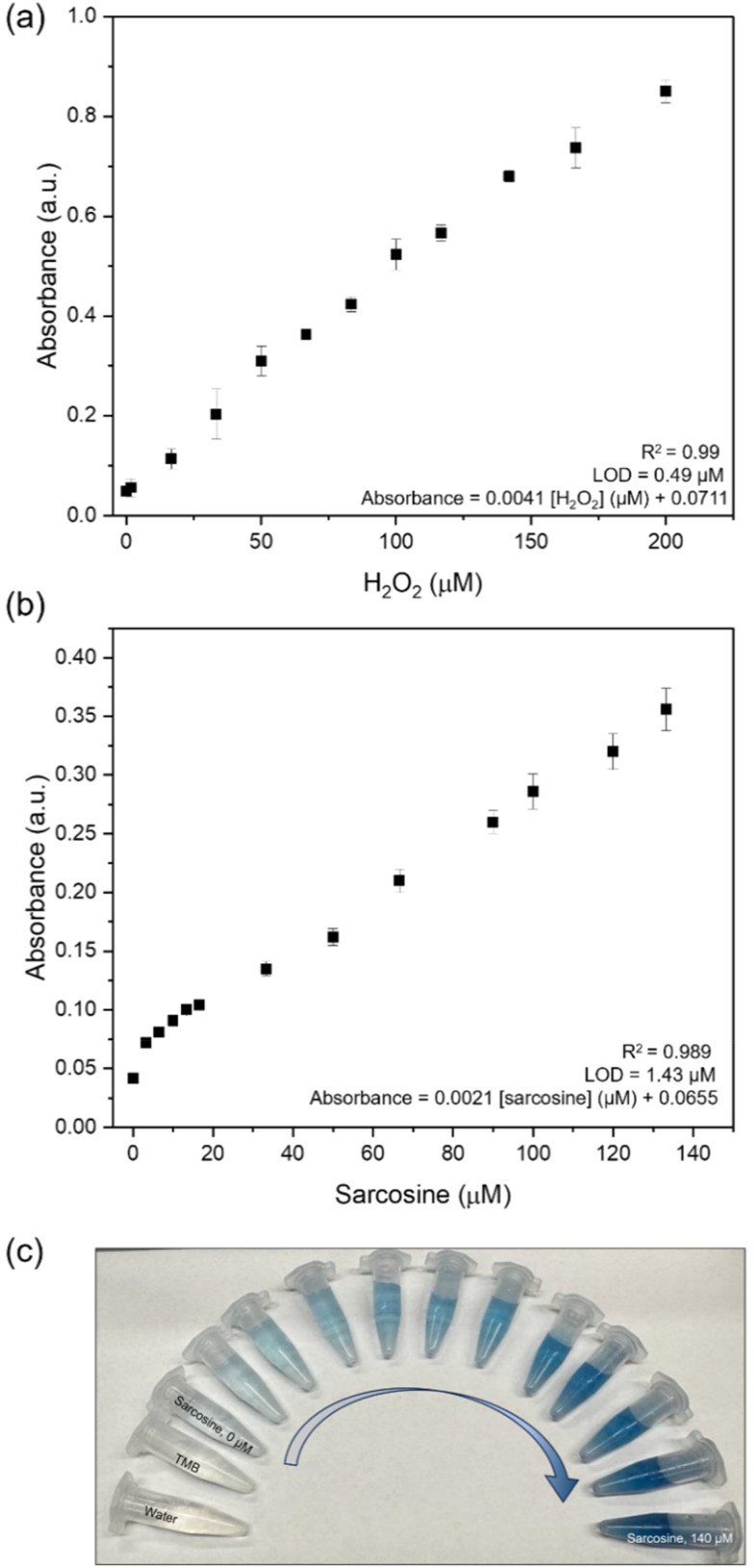
Detection of H_2_O_2_ and sarcosine. (a) Linear calibration curves for varying concentrations of H_2_O_2_ with TMB at 1 mM, and (b) varying concentrations of sarcosine using sarcosine oxidase and Cu nanozymes with TMB at 1 mM. Error bars are standard deviations from three replicate measurements. (c) Corresponding images of visible color in the presence of varying concentrations of sarcosine.

We then established an H_2_O_2_ colorimetric sensor using the optimized reaction conditions. A calibration curve for measurement of H_2_O_2_ was created by adding different concentrations of H_2_O_2_ in 200 μL of 1 mM TMB in the presence of Cu nanocubes (Fig. 4a). Absorbance at 652 nm was recorded after 30 minutes; the resulting linear calibration equation was:*A*_652_ = 0.0041[H_2_O_2_] + 0.0711with the concentration of H_2_O_2_ in μM and yielding an *R*^2^ value of 0.99. The limit of detection (LOD) was determined using the formula: LOD = 3*σ*/*m*, where *σ* represents the relative standard deviation and *m* is the slope from the linear plot in Fig. 4(a). The LOD was calculated as 0.49 μM with a linear dynamic range of 1.6 to 200 μM. This indicates that Cu nanocubes exhibited good sensing performance for H_2_O_2_ with notable sensitivity (0.0041 μM^−1^) and a reasonable LOD compared to previously reported values (Table 2). The creation of Cu^2+^ on the etched copper may have played a key role in enhancing H_2_O_2_ adsorption or electron transfer, yielding a viable nanozyme.

**Table 2:** Comparison of H_2_O_2_ detection in different colorimetric systems

Material	Linear range (μM)	LOD (μM)	Ref.
FePt-Au HNPs	20–700	12.33	34
Cu_2_(OH)_3_Cl-CeO_2_	20–50	10	35
Fe_3_O_4_@Cu@Cu_2_O	4000–50 000	2000	36
N-G-Fe_3_O_4_	0–10 000	17.3	37
GQDs/AgNPs	0.1–100	0.033	38
AgNPs	0.05–7.5	0.032	39
Fe-doped g-C_3_N_4_	2–100	1.8	40
Fe-CoO NCs	6–20	4.4	41
FeS_2_ NPs	2–80	0.91	42
Carbon quantum dots	5–60	0.86	43
Co-doped CuS	10–100	2.2	44
b-TiO_2_/29mTHPP	5–500	1	45
SiO_2_@TiO2/PDI-OH	0–400	0.076	46
Cu nanocubes	1.6–200	0.49	This work

A sarcosine sensor was then implemented by creating a cascading reaction system coupling the Sox-mediated aerobic oxidation of sarcosine to Cu nanocube-mediated oxidation of TMB. Since the concentration of the H_2_O_2_ generated by Sox is proportional to the concentration of sarcosine, the resulting absorbance intensity serves as an indirect indicator of sarcosine concentration. Fig. 4b shows the calibration curve for sarcosine concentrations, with the corresponding visual readouts of colorimetric changes in Fig. 4c. Absorbance at 652 nm was recorded after 30 minutes of nanozyme reaction; the resulting linear calibration equation was:*A*_652_ = 0.0021[sarcosine] + 0.0655with the concentration of sarcosine in μM. The calibration curve yielded an *R*^2^ = 0.989 in the linear range of 0–140 μM, and a LOD and LOQ of 1.43 μM and 4.7 μM, respectively. Notably, the LOD of the proposed method matched well with the reference range for serum sarcosine concentrations, making it potentially suitable for clinical applications and competitive with previously reported approaches (Table 3). The calibration equation for sarcosine aligns well with the one obtained for the H_2_O_2_ sensing.

**Table 3:** Comparison of sarcosine detection in different colorimetric systems

Materials	Linear range (uM)	LOD (uM)	Ref.
NQs/GO	6.2–263	0.73	8
PteFe3O4@C/GCE	0.5–60	0.43	63
Pt@ZIF8/GCE	5–30	1.06	64
CNT/Pt	6–750	6	65
Silver solid amalgam electrode	7.5–500	2	66
BCD + MnO_2_ NSs + OPD	1–80	0.36	67
PVA-Au-pph TEOS-SOD-GE	500–7500	500	68
SiO_2_@TiO2/PDI-OH	0.2–400	0.076	69
FePt-Au HNPs	20–700	12.33	70
Cu_2_(OH)_3_Cl-CeO_2_	20–50	10	71 and 72
Fe_3_O_4_@Cu@Cu_2_O	4000–50 000	2000	72
N-G-Fe3O4	0–10 000	17.3	73
GQDs/AgNPs	0.1–100	0.033	74
AgNPs	0.05–7.5	0.032	75
Fe-doped g-C_3_N_4_	2–100	1.8	76
Fe-CoO NCs	6–20	4.4	77
FeS_2_ NPs	2–80	0.91	78
Carbon quantum dots	5–60	0.86	79
Co-doped CuS	10–100	2.2	80
h-Fe_3_O_4_@ppy	0.2–100	0.18	81
b-TiO_2_/29mTHPP	5–500	1	82
Cu nanocubes	3.3–140	1.43	This work

**Fig. 5:**
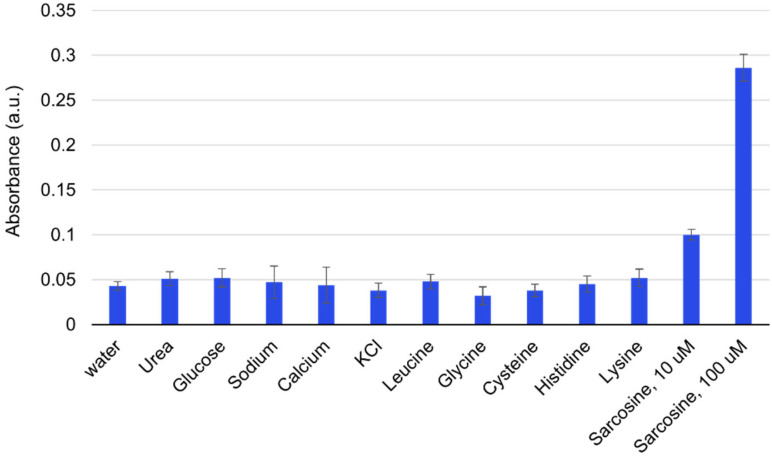
Selectivity of the Cu nanozyme sensing platform. The concentration of all compounds was 100 μM except for the one experiment with sarcosine at 10 μM as indicated in the graph. All error bars were estimated from three replicate measurements.

To evaluate the selectivity of the colorimetric sensor for sarcosine, we studied the influence of potential interfering substances on sarcosine quantification. Potential high-concentration interferents including urea, glucose, sodium, and potassium were tested, as well as structurally similar molecules including glycine (different from sarcosine by only one methyl group) and other amino acids including leucine, cysteine, and histidine. These potential interferents were added at 100 μM into reactions containing Sox TMB, and Cu nanocubes. As shown in Fig. 5, an absorbance signal was only detected for sarcosine, due to the high specificity of sarcosine oxidase.

### Stability and reusability of Cu nanostructured thin films

**Fig. 6:**
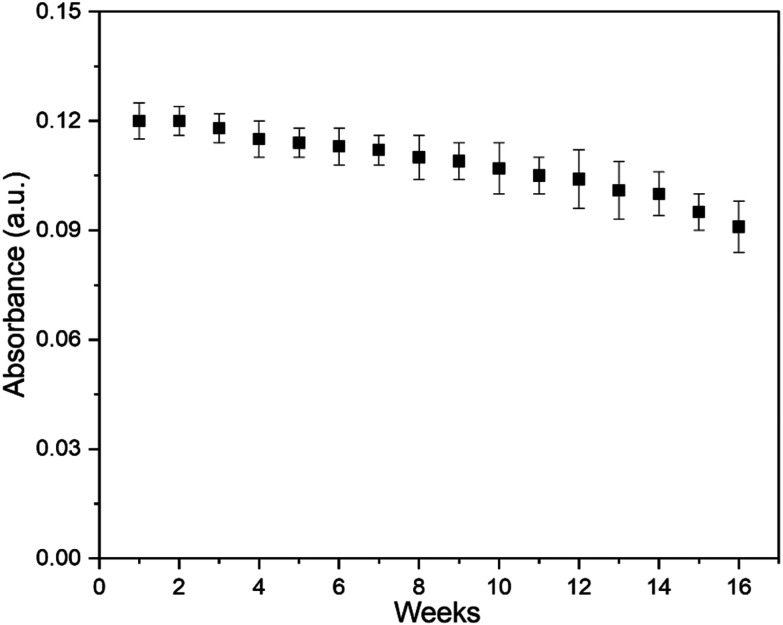
Absorbance of oxidized TMB solutions at 652 nm for 16 weeks. For every data point, a fresh solution of TMB was prepared and reacted in the presence of Cu nanozymes and 10 μM H_2_O_2_ for 30 minutes. The nanocubes were stored in water after each use to avoid oxidation. Error bars represent the standard deviation of three replicate measurements.

We then assessed the stability and reusability of Cu nanocubes in serial reactions using the same nanocubes. After each reaction cycle and sarcosine measurement, the catalytic film was removed from the solution, rinsed, and immersed in water for storage until performing the next measurement one week later. As shown in Fig. 6, nanocube function remained relatively consistent from reaction to reaction, with only 20.8% loss of activity after 16 cycles over four months. This decrease in activity may be attributed to adsorbed biomolecules from the complex reaction mixtures potentially blocking some active sites. Nonetheless, the maintained function after months of weekly assays demonstrates excellent reusability for this nanomaterial. In addition, since the nanocubes are surface-anchored and can be easily removed from the reaction, the use of a reaction-terminating agent—often used in colorimetric TMB assays to halt reactions to make measurements more reproducible—is no longer necessary, making the use of Cu nanocubes simpler than many other nanomaterials. Overall, these results underscore the potential of Cu nanocubes to be environmentally friendly, cost-effective, and robust enough for use in practical applications. The stability of surface-anchored Cu nanocubes even across months of reactions suggests the potential for use in a device with reusable nanozyme, reducing the per-assay cost of reagents or enabling the implementation of sample-specific calibration to account for matrix effects.^83^

### Quantification of sarcosine in serum

To assess the biosensor's performance in a context closer to practical applications, we tested it using spiked serum samples. Known amounts of sarcosine starting from the healthy range in human serum^2^ (1.4 ± 0.6 μM) and into the pathological range were added to mouse serum to determine the recovery rate in the presence of other interfering compounds. The absorbance of blue oxTMB was recorded using a plate reader, and the concentration of sarcosine was calculated using the previously obtained linear calibration equation. Table 4 shows that even in this complex sample, sarcosine levels were measured effectively in both the normal and pathological ranges, with good recovery and high repeatability.

**Table 4:** Recovery of sarcosine in serum samples

Sample spiked (μM)	Detected (μM)	Recovery (%)	RSD of recovery (%)
2	1.88	94	
2	1.78	89	2.8
2	1.82	91	
5	4.63	92.6	
5	4.71	94.2	1.3
5	4.59	91.8	
10	9.52	95.2	
10	9.68	96.8	2.9
10	9.15	91.5	
15	14.65	94.9	
15	14.49	96.6	0.9
15	14.74	95.6	
20	19.36	96.8	
20	19.74	96.2	0.5
20	19.68	95.9	

### Prototype for low-cost, automatic, unambiguous interpretation

As noted above, existing methods for sarcosine measurement are not compatible with point-of-care use in resource-limited settings, where inexpensive diagnostics (even if semi-quantitative) can have a substantial impact. To address this challenge, we designed and implemented the prototype for an economical and user-friendly point-of-care device using a microcontroller, LEDs, and a light-dependent resistor (LDR) to report semi-quantitative sarcosine levels (Fig. S4†). The LDR was programmed (code given in ESI†) to light up specific LEDs in response to the detection of different absorbance intensities corresponding to different sarcosine concentrations. ESI Movie 1† shows the LED responses to different samples, demonstrating the prototype's effectiveness in minimal-equipment and deployable readout of sarcosine concentrations: 0 μM turned on no LEDs, 5 μM turned on one red LED, 50 μM turned on two red LEDs, and 100 μM turned on all LEDs. This device showcases the adaptability of the nanozyme-based sarcosine biosensor, integrating simple electrical components to produce a user-friendly assay requiring minimal training.

## Conclusion

In summary, we have developed an inexpensive electrochemical etching approach to generate immobilized films of Cu nanocubes, finding that these films exhibited peroxidase-like activity to catalyze the oxidation of TMB in place of horseradish peroxidase. We used this nanozyme to develop a colorimetric sensor for sarcosine detection using cascading reactions; this strategy shows good performance across a wide linear range (0–140 μM) with a low LOD (1.43 μM) and LOQ (4.7 μM). The low synthesis cost, good reusability and stability, high selectivity, excellent analytical performance, and visually interpretable detection suggest that Cu nanocube-based sensors may be a promising tool for sarcosine detection. This work not only broadens the choice of nanozymes available, but also provides a one-pot, peroxidase-free strategy for the colorimetric detection of sarcosine. While this strategy still uses one enzyme coupled with the nanozyme, it is still a desirable alternative to using two enzymes. Maintaining function during storage and identifying ideal reaction conditions for just one enzyme is much more straightforward than managing those requirements for two enzymes simultaneously, making the replacement of just one enzyme (horseradish peroxidase) with nanozymes a valuable advance. Moreover, the simplicity of the copper etching strategy leads to a low-cost, straightforward synthesis that is preferable to an additional enzyme purification. In the future, characterization of the etched Cu nanocubes’ ability to mimic different enzymes’ catalytic activities (such as catalase to convert H_2_O_2_ into water and oxygen, or oxidase) would help to more completely flesh out the potential application space for this nanomaterial; exploring other different nanonzymes that can achieve those activities could even further help the nanozyme-based approach to address the limitations of enzymes.

## Experimental

### Materials

CuCl_2_, TMB, sodium acetate, acetic acid, sarcosine, and sarcosine oxidase enzyme were purchased from Sigma-Aldrich. H_2_O_2_ and Cu foil were purchased from ThermoFisher. Insulating tape was purchased from 3 M. Organic solvents acetone (99.5%), methanol (99.8%), and isopropanol (99.5%) were purchased from VWR International.

### Synthesis of Cu nanostructured thin film

Two Cu foil samples of different dimensions (4 × 4 cm^2^ and 5 × 5 cm^2^) were prepared by cutting Cu foil from stock. These samples were designated as the working and counter electrodes, respectively. Before electrochemical surface modification with a DC power supply, the samples underwent a cleaning process with acetone, methanol, and isopropanol to eliminate organic contaminants. They were then air-dried at room temperature. Electrical connections between steel wire and the Cu foil samples were established by masking the Cu surface with insulating tape. Electrochemical surface modification took place with a power supply set at 8 volts and 4 amperes for various time intervals in 1 M CuCl_2_ solution. The distance between the working and counter electrodes was 3 cm. Following electrochemical etching, the sample was taken out from the electrochemical cell, washed with deionized water, and dried at room temperature. The Cu foil was then cut into 1 × 0.5 cm^2^ pieces for characterization and subsequent use for sensing purposes.

### Evaluation of peroxidase-like activity

Peroxidase-like activity of Cu nanocubes was evaluated by measuring the absorbance of oxTMB solution after contact between the Cu nanocube foil and a solution containing TMB and H_2_O_2_. Briefly, a Cu nanocube film of 0.5 ± 0.02 cm^2^ size was submerged in a 500 μL solution containing 2 mM TMB and varying concentrations of H_2_O_2_. Cu nanocubes catalyze the TMB-H_2_O_2_ reaction to form blue oxTMB solution. After 15 minutes of contact, the film was removed, and the solution's absorbance was measured using a SYNERGY BioTek plate reader at 652 nm to allow estimation of initial reaction velocity. Reaction kinetic parameters were calculated by fitting Lineweaver–Burk double-reciprocal plots. The Michaelis–Menten constant (*K*_m_) and the maximum reaction velocity (*V*_max_) were calculated using the following equation:1
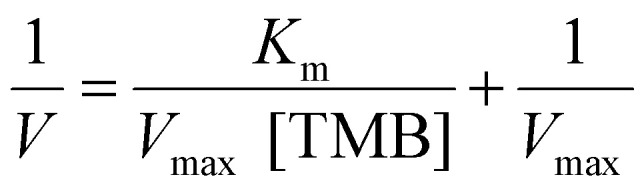
where *V* is the initial reaction velocity and [TMB] refers to the substrate concentration. The concentrations and reaction rates were extracted from the absorbance data according to Beer–Lambert's law, *A* = *ε*l[TMB], where *A* is the absorbance at 652 nm, *ε* = 3.9 × 10^4^ M^−1^ cm^−1^ is the extinction coefficient for oxTMB, and l is the path length (*i.e.*, 1 cm).

### Colorimetric assay for hydrogen peroxide and sarcosine

150 μL of varying concentrations of sarcosine containing 50 μL of sarcosine oxidase (1.8 g mL^−1^ at pH 7.5) was added to a 2 mL centrifuge tube and incubated at 37 °C for 1 h after mixing thoroughly. Next, TMB (250 μL, 1 mM) solution, an acetic acid/sodium acetate buffer solution (1050 μL, 0.1 M), and 0.5 cm^2^ Cu etched foil were added to the centrifuge tube which was incubated for 30 min in a constant temperature water bath at room temperature. For H_2_O_2_ sensing, 150 μL of varying concentrations of H_2_O_2_ was added to 250 μL of 1 mM TMB solution and 0.5 cm^2^ Cu etched foil. 1100 μL of buffer solution was added to make a total solution volume of 1500 μL. The absorbance at 652 nm was recorded. The limit of detection (LOD) and limit of quantification (LOQ) were calculated based on the formulas LOD = 3*σ*/*m* and LOQ = 3.3 × LOD, where *σ* is the standard deviation of the absorbance measurement of the blank (calculated based on four measurements of independent samples), and m is the slope of the absorbance *vs.* concentration curve.^84^

### Reusability and complex matrix recovery assessments

The reusability of the Cu nanocubes was assessed by recording absorbance spectra following 1 mM TMB/10 μM H_2_O_2_ reactions executed once a week for 16 weeks. Each reaction cycle lasted for 30 minutes with the same Cu nanocubes. After each measurement, the catalytic film was removed from the solution, rinsed, and stored in water before being immersed in a fresh TMB/H_2_O_2_ solution to collect the next absorbance at 652 nm.

To test sensors response in a complex matrix, serum was collected from mouse (BALB/c strain, IACUCA100576-08/16/2025). All samples were diluted at 1 : 1 with 0.1 M acetate buffer. To assess sarcosine recovery, 150 μL of the known concentration of sarcosine was added to 500 μL diluted serum and incubated with 50 μL of sarcosine oxidase for 1 hour before mixing them with 250 μL of 1 mM TMB solution with Cu nanocubes. The oxTMB solution absorbance was recorded and compared with the linear calibration curve. % recovery was calculated using the formula: (spiked sample-unspiked sample)/(spiked sample).

### Device prototype

Our custom prototype setup features an Arduino system with a microcontroller, five 10 K Ohm resistors, four 5V LEDs, and a light-dependent resistor (LDR). We placed 500 μl solutions with varying sarcosine concentrations (0 μM, 5 μM, 50 μM, and 100 μM) in a holder on the breadboard. The LDR responds to the light intensity reaching it, which is programmed to detect varying light intensities and activate specific LEDs corresponding to different sarcosine concentrations.

## Author contributions

Anuja Tripathi: conceptualization, visualization, methodology, formal analysis, investigation, and writing – original draft. Mark P. Styczynski: conceptualization, writing – review & editing, and supervision.

## Data availability

The data supporting this article have been included in the ESI†. The code used for the prototype device is openly available in Github at https://github.com/gtStyLab/nanozymeLdrLed.git.

## Conflicts of interest

There are no conflicts to declare.

## Supplementary Material

AN-150-D4AN01242A-s001

AN-150-D4AN01242A-s002

## References

[cit1] Sreekumar A., Poisson L. M., Rajendiran T. M., Khan A. P., Cao Q., Yu J., Laxman B., Mehra R., Lonigro R. J., Li Y., Nyati M. K., Ahsan A., Kalyana-Sundaram S., Han B., Cao X., Byun J., Omenn G. S., Ghosh D., Pennathur S., Alexander D. C., Berger A., Shuster J. R., Wei J. T., Varambally S., Beecher C., Chinnaiyan A. M. (2009). Metabolomic Profiles Delineate Potential Role for Sarcosine in Prostate Cancer Progression. Nature.

[cit2] Kumar P., Narwal V., Jaiwal R., Pundir C. S. (2018). Construction and Application of Amperometric Sarcosine Biosensor Based on SOxNPs/AuE for Determination of Prostate Cancer. Biosens. Bioelectron..

[cit3] Wang L., Liu S., Yang W., Yu H., Zhang L., Ma P., Wu P., Li X., Cho K., Xue S., Jiang B. (2017). Plasma Amino Acid Profile in Patients with Aortic Dissection. Sci. Rep..

[cit4] Munshi S. U., Rewari B. B., Bhavesh N. S., Jameel S. (2013). Nuclear Magnetic Resonance Based Profiling of Biofluids Reveals Metabolic Dysregulation in HIV-Infected Persons and Those on Anti-Retroviral Therapy. PLoS One.

[cit5] Piert M., Shao X., Raffel D., Davenport M. S., Montgomery J., Kunju L. P., Hockley B. G., Siddiqui J., Scott P. J. H., Chinnaiyan A. M., Rajendiran T. (2017). Preclinical Evaluation of 11 C-Sarcosine as a Substrate of Proton-Coupled Amino Acid Transporters and First Human Application in Prostate Cancer. J. Nucl. Med..

[cit6] Spence D. (2012). Does Early Diagnosis Really Save Lives?. Br. Med. J..

[cit7] Mello L. D., Kubota L. T. (2002). Review of the Use of Biosensors as Analytical Tools in the Food and Drink Industries. Food Chem..

[cit8] Xue Z., Yin B., Wang H., Li M., Rao H., Liu X., Zhou X., Lu X. (2016). An Organic Indicator Functionalized Graphene Oxide Nanocomposite-Based Colorimetric Assay for the Detection of Sarcosine. Nanoscale.

[cit9] Lee J., Liao H., Wang Q., Han J., Han J.-H., Shin H. E., Ge M., Park W., Li F. (2022). Exploration of Nanozymes in Viral Diagnosis and Therapy. Exploration.

[cit10] Huang L., Chen J., Gan L., Wang J., Dong S. (2019). Single-Atom Nanozymes. Sci. Adv..

[cit11] Liang M., Yan X. (2019). Nanozymes: From New Concepts, Mechanisms, and Standards to Applications. Acc. Chem. Res..

[cit12] Gooding J. J. (2019). Can Nanozymes Have an Impact on Sensing?. ACS Sens..

[cit13] Wei H., Wang E. (2013). Nanomaterials with Enzyme-like Characteristics (Nanozymes): Next-Generation Artificial Enzymes. Chem. Soc. Rev..

[cit14] Wu J., Wang X., Wang Q., Lou Z., Li S., Zhu Y., Qin L., Wei H. (2019). Nanomaterials with Enzyme-like Characteristics (Nanozymes): Next-Generation Artificial Enzymes (II). Chem. Soc. Rev..

[cit15] Shang C., Wang Q., Tan H., Lu S., Wang S., Zhang Q., Gu L., Li J., Wang E., Guo S. (2022). Defective PtRuTe As Nanozyme with Selectively Enhanced Peroxidase-like Activity. JACS Au.

[cit16] Tripathi A., Harris K. D., Elias A. L. (2021). High Surface Area Nitrogen-Functionalized Ni Nanozymes for Efficient Peroxidase-like Catalytic Activity. PLoS One.

[cit17] Tripathi A., Harris K. D., Elias A. L. (2020). Peroxidase-Like Behavior of Ni Thin Films Deposited by Glancing Angle Deposition for Enzyme-Free Uric Acid Sensing. ACS Omega.

[cit18] Canonica F., Klose D., Ledermann R., Sauer M. M., Abicht H. K., Quade N., Gossert A. D., Chesnov S., Fischer H.-M., Jeschke G., Hennecke H., Glockshuber R. (2019). Structural Basis and Mechanism for Metallochaperone-Assisted Assembly of the Cu A Center in Cytochrome Oxidase. Sci. Adv..

[cit19] Li X., Qiu S., Shi J., Wang S., Wang M., Xu Y., Nie Z., Liu C., Liu C. (2019). A New Function of Copper Zinc Superoxide Dismutase: As a Regulatory DNA-Binding Protein in Gene Expression in Response to Intracellular Hydrogen Peroxide. Nucleic Acids Res..

[cit20] Muthuvelu K. S., Rajarathinam R., Selvaraj R. N., Rajendren V. B. (2020). A Novel Method for Improving Laccase Activity by Immobilization onto Copper Ferrite Nanoparticles for Lignin Degradation. Int. J. Biol. Macromol..

[cit21] Wei G., Liu S., Peng Y., Wei H. (2024). On the Specificity of Nanozymes: A Perspective. Chin. J. Chem..

[cit22] Feng J., Yao T., Ma Z. (2023). Recent Advances of Peroxidase-Active Nanozymes in Electrochemical Immunoassays. Sens. Diagn..

[cit23] Qu L., Han J., Huang Y., Yang G., Liu W., Long Z., Gu Y., Zhang Q., Gao M., Dong X. (2023). Peroxidase-like Nanozymes for Point-of-Care SERS Sensing and Wound Healing. ACS Appl. Bio. Mater..

[cit24] Dong H., Du W., Dong J., Che R., Kong F., Cheng W., Ma M., Gu N., Zhang Y. (2022). Depletable Peroxidase-like Activity of Fe3O4 Nanozymes Accompanied with Separate Migration of Electrons and Iron Ions. Nat. Commun..

[cit25] Zhao L., Wu Z., Liu G., Lu H., Gao Y., Liu F., Wang C., Cui J., Lu G. (2019). High-Activity Mo, S Co-Doped Carbon Quantum Dot Nanozyme-Based Cascade Colorimetric Biosensor for Sensitive Detection of Cholesterol. J. Mater. Chem. B.

[cit26] Wei H., Wang E. (2008). Fe_3_O_4_ Magnetic Nanoparticles as Peroxidase Mimetics and Their Applications in H_2_O_2_ and Glucose Detection. Anal. Chem..

[cit27] Wang M., Jiang M., Luo X., Zhang L., He Y., Xue F., Su X. (2024). High-Performance Colorimetric Sensor Based on PtRu Bimetallic Nanozyme
for Xanthine Analysis. Food Chem X.

[cit28] Wang K., Hong Q., Zhu C., Xu Y., Li W., Wang Y., Chen W., Gu X., Chen X., Fang Y., Shen Y., Liu S., Zhang Y. (2024). Metal-Ligand Dual-Site Single-Atom Nanozyme Mimicking Urate Oxidase with High Substrates Specificity. Nat. Commun..

[cit29] Kwon S., Zhang J., Ganganahalli R., Verma S., Yeo B. S. (2023). Enhanced Carbon Monoxide Electroreduction to >1 A Cm^−2^ C_2+_ Products Using Copper Catalysts Dispersed on MgAl Layered Double Hydroxide Nanosheet House–of–Cards Scaffolds. Angew. Chem., Int. Ed..

[cit30] Gao L., Zhuang J., Nie L., Zhang J., Zhang Y., Gu N., Wang T., Feng J., Yang D., Perrett S., Yan X. (2007). Intrinsic Peroxidase-like Activity of Ferromagnetic Nanoparticles. Nat. Nanotechnol..

[cit31] Wang D., Song X., Li P., Gao X. J., Gao X. (2020). Origins of the Peroxidase Mimicking Activities of Graphene Oxide from First Principles. J. Mater. Chem. B.

[cit32] Wang C., Long Y., Deng Y., Han Y., Tishkevich D., Ha M. N., Weng Q. (2024). Hexagonal Boron Nitride Nanomaterials for Biomedical Applications. BMEMat.

[cit33] Zhao X., Li S., Yu X., Gang R., Wang H. (2020). *In Situ* Growth of CeO_2_ on g-C_3_N_4_ Nanosheets toward a Spherical g-C_3_N_4_/CeO_2_ Nanozyme with Enhanced Peroxidase-like Catalysis: A Selective Colorimetric Analysis Strategy for Mercury (II). Nanoscale.

[cit34] Maity T., Jain S., Solra M., Barman S., Rana S. (2022). Robust and Reusable Laccase Mimetic Copper Oxide Nanozyme for Phenolic Oxidation and Biosensing. ACS Sustain. Chem. Eng..

[cit35] Wu Y., Wu J., Jiao L., Xu W., Wang H., Wei X., Gu W., Ren G., Zhang N., Zhang Q., Huang L., Gu L., Zhu C. (2020). Cascade Reaction System Integrating Single-Atom Nanozymes with Abundant Cu Sites for Enhanced Biosensing. Anal. Chem..

[cit36] Shao J., Josephs E. A., Lee C., Lopez A., Ye T. (2013). Electrochemical Etching of Gold within Nanoshaved Self-Assembled Monolayers. ACS Nano.

[cit37] Mardiansyah D., Badloe T., Triyana K., Mehmood M. Q., Raeis-Hosseini N., Lee Y., Sabarman H., Kim K., Rho J. (2018). Effect of Temperature on the Oxidation of Cu Nanowires and Development of an Easy to Produce, Oxidation-Resistant Transparent Conducting Electrode Using a PEDOT : PSS Coating. Sci. Rep..

[cit38] Dong Y., Wang K., Tan Y., Wang Q., Li J., Mark H., Zhang S. (2018). Synthesis and Characterization of Pure Copper Nanostructures Using Wood Inherent Architecture as a Natural Template. Nanoscale Res. Lett..

[cit39] Hussein H. M. (2023). Fabricating and Synthesizing Spin Coated CuO Thin Film as Absorber Layer in Optoelectronic Applications. Prot. Met. Phys. Chem. Surf..

[cit40] Fentahun D. A., Tyagi A., Singh S., Sinha P., Mishra A., Danayak S., Kumar R., Kar K. K. (2021). Tunable Optical and Electrical Properties of P-Type Cu2O Thin Films. J. Mater. Sci.: Mater. Electron..

[cit41] Liu B.-H., Huber M., van Spronsen M. A., Salmeron M., Bluhm H. (2022). Ambient Pressure X-Ray Photoelectron Spectroscopy Study of Room-Temperature Oxygen Adsorption on Cu(1 0 0) and Cu(1 1 1). Appl. Surf. Sci..

[cit42] Zhou J. (2004). Catalytic Oxidation of Pyridine on the Supported Copper Catalysts in the Presence of Excess Oxygen. J. Catal..

[cit43] Peng B., Liang S., Yan Z., Wang H., Meng Z., Zhang M. (2022). Generation of Multi-Valence Cu_x_ O by Reduction with Activated Semi-Coke and Their Collaboration in the Selective Reduction of NO with NH_3_. RSC Adv..

[cit44] Qin P., Lei H., Zheng X., Liu Q., Tao H., Yang G., Ke W., Xiong L., Qin M., Zhao X., Fang G. (2016). Copper–Doped Chromium Oxide Hole–Transporting Layer for Perovskite Solar Cells: Interface Engineering and Performance Improvement. Adv. Mater. Interfaces.

[cit45] Zhou J. (2004). Catalytic Oxidation of Pyridine on the Supported Copper Catalysts in the Presence of Excess Oxygen. J. Catal..

[cit46] Sielska A., Cembrowska-Lech D., Kowalska-Góralska M., Czerniawski R., Krepski T., Skuza L. (2024). Effects of Copper Nanoparticles on Oxidative Stress Genes and Their Enzyme Activities in Common Carp (*Cyprinus Carpio*). Eur. Zool. J..

[cit47] Illakkia R., Mahesh N., Balakumar S., Sivakumar N., Shree G. G. K., Rajan A. P., Govindasamy C., Aravind J. (2024). Adroit Effect of Copper Nanoparticles and Copper Nanozyme and Their Effective Decolorization of Azo Dyes. J. King Saud Univ. Sci..

[cit48] Chen F., Liu L., Wu J., Rui X., Chen J., Yu Y. (2022). Single–Atom Iron Anchored Tubular G–C_3_ N_4_ Catalysts for Ultrafast Fenton–Like Reaction: Roles of High–Valency Iron–Oxo Species and Organic Radicals. Adv. Mater..

[cit49] Zhou Z., Wang Y., Peng F., Meng F., Zha J., Ma L., Du Y., Peng N., Ma L., Zhang Q., Gu L., Yin W., Gu Z., Tan C. (2022). Intercalation–Activated Layered MoO _3_ Nanobelts as Biodegradable Nanozymes for Tumor–Specific Photo–Enhanced Catalytic Therapy. Angew. Chem., Int. Ed..

[cit50] Wang N., Li B., Qiao F., Sun J., Fan H., Ai S. (2015). Humic Acid-Assisted Synthesis of Stable Copper Nanoparticles as a Peroxidase Mimetic and Their Application in Glucose Detection. J. Mater. Chem. B.

[cit51] Das B., Lou-Franco J., Gilbride B., Ellis M. G., Stewart L. D., Grant I. R., Balasubramanian P., Cao C. (2022). Peroxidase-Mimicking Activity of Biogenic Gold Nanoparticles Produced from *Prunus Nepalensis* Fruit Extract: Characterizations and Application for the Detection of *Mycobacterium Bovis*. ACS Appl. Bio Mater..

[cit52] Liu C., Tan L., Zhang K., Wang W., Ma L. (2023). Immobilization of Horseradish Peroxidase for Phenol Degradation. ACS Omega.

[cit53] Wu X., Chen T., Wang J., Yang G. (2018). Few-Layered MoSe_2_ Nanosheets as an Efficient Peroxidase Nanozyme for Highly Sensitive Colorimetric Detection of H_2_O_2_ and Xanthine. J. Mater. Chem. B.

[cit54] Xie X., Chen X., Wang Y., Zhang M., Fan Y., Yang X. (2023). High-Loading Cu Single-Atom Nanozymes Supported by Carbon Nitride with Peroxidase-like Activity for the Colorimetric Detection of Tannic Acid. Talanta.

[cit55] Wang J., Hu Y., Zhou Q., Hu L., Fu W., Wang Y. (2019). Peroxidase-like Activity of Metal–Organic Framework [Cu(PDA)(DMF)] and Its Application for Colorimetric Detection of Dopamine. ACS Appl. Mater. Interfaces.

[cit56] Peng L.-J., Zhou H.-Y., Zhang C.-Y., Yang F.-Q. (2022). Study on the Peroxidase-like Activity of Cobalt Phosphate and Its Application in Colorimetric Detection of Hydrogen Peroxide. Colloids Surf. A: Physicochem. Eng. Asp..

[cit57] Cheng R., Xiao Z., Tang X., Xu P., Qiu P. (2025). Nickel-Doped Cuprous Oxide Nanocauliflowers with Specific Peroxidase-like Activity for Sensitive Detection of Hydrogen Peroxide and Uric Acid. Colloids Surf., B.

[cit58] Wu Y., Wu J., Jiao L., Xu W., Wang H., Wei X., Gu W., Ren G., Zhang N., Zhang Q., Huang L., Gu L., Zhu C. (2020). Cascade Reaction System Integrating Single-Atom Nanozymes with Abundant Cu Sites for Enhanced Biosensing. Anal. Chem..

[cit59] Zhang X.-Q., Gong S.-W., Zhang Y., Yang T., Wang C.-Y., Gu N. (2010). Prussian Blue Modified Iron Oxide Magnetic Nanoparticles and Their High Peroxidase-like Activity. J. Mater Chem..

[cit60] Liu H., Ding Y., Yang B., Liu Z., Liu Q., Zhang X. (2018). Colorimetric and Ultrasensitive Detection of H_2_O_2_ Based on Au/Co3O4-CeOx Nanocomposites with Enhanced Peroxidase-like Performance. Sens. Actuators B Chem.

[cit61] Jampaiah D., Reddy T. S., Kandjani A. E., Selvakannan P. R., Sabri Y. M., Coyle V. E., Shukla R., Bhargava S. K. (2016). Fe-Doped CeO_2_ Nanorods for Enhanced Peroxidase-like Activity and Their Application towards Glucose Detection. J. Mater. Chem. B.

[cit62] Peng L.-J., Zhou H.-Y., Zhang C.-Y., Yang F.-Q. (2022). Study on the Peroxidase-like Activity of Cobalt Phosphate and Its Application in Colorimetric Detection of Hydrogen Peroxide. Colloids Surf. A: Physicochem. Eng. Asp..

[cit63] Yang Q., Li N., Li Q., Chen S., Wang H.-L., Yang H. (2019). Amperometric Sarcosine Biosensor Based on Hollow Magnetic Pt–Fe3O4@C Nanospheres. Anal. Chim. Acta.

[cit64] Yang H., Wang J., Yang C., Zhao X., Xie S., Ge Z. (2018). Nano Pt@ZIF8 Modified Electrode and Its Application to Detect Sarcosine. J. Electrochem. Soc..

[cit65] Pannell M. J., Doll E. E., Labban N., Wayu M. B., Pollock J. A., Leopold M. C. (2018). Versatile Sarcosine and Creatinine Biosensing Schemes Utilizing Layer-by-Layer Construction of Carbon Nanotube-Chitosan Composite Films. J. Electroanal. Chem..

[cit66] Josypčuk O., Barek J., Josypčuk B. (2015). Construction and Application of Flow Enzymatic Biosensor Based of Silver Solid Amalgam Electrode for Determination of Sarcosine. Electroanalysis.

[cit67] Li W., Li T., Chen S., Deng D., Ji Y., Li R. (2022). Nanozyme-Mediated Cascade Reaction System for Ratiometric Fluorescence Detection of Sarcosine. Sens. Actuators, B.

[cit68] Lad U., Kale G. M., Bryaskova R. (2014). Sarcosine Oxidase Encapsulated Polyvinyl Alcohol-Silica-AuNP Hybrid Films for Sarcosine Sensing Electrochemical Bioelectrode. J. Electrochem. Soc..

[cit69] Liu Q., Cao S., Sun Q., Xing C., Gao W., Lu X., Li X., Yang G., Yu S., Chen Y. (2022). A Perylenediimide Modified SiO_2_@TiO2 Yolk-Shell Light-Responsive Nanozyme: Improved Peroxidase-like Activity for H_2_O_2_ and Sarcosine Sensing. J. Hazard. Mater..

[cit70] Ding Y., Yang B., Liu H., Liu Z., Zhang X., Zheng X., Liu Q. (2018). FePt-Au Ternary Metallic Nanoparticles with the Enhanced Peroxidase-like Activity for Ultrafast Colorimetric Detection of H_2_O_2_. Sens. Actuators, B.

[cit71] Wang N., Sun J., Chen L., Fan H., Ai S. (2015). A Cu2(OH)3Cl-CeO_2_ Nanocomposite with Peroxidase-like Activity, and Its Application to the Determination of Hydrogen Peroxide, Glucose and Cholesterol. Microchim. Acta.

[cit72] Wang Z., Chen M., Shu J., Li Y. (2016). One-Step Solvothermal Synthesis of Fe3O4@Cu@Cu2O Nanocomposite as Magnetically Recyclable Mimetic Peroxidase. J. Alloys Compd..

[cit73] Zhang W., Chen C., Yang D., Dong G., Jia S., Zhao B., Yan L., Yao Q., Sunna A., Liu Y. (2016). Optical Biosensors Based on Nitrogen–Doped Graphene Functionalized with Magnetic Nanoparticles. Adv. Mater. Interfaces.

[cit74] Lin T., Zhong L., Wang J., Guo L., Wu H., Guo Q., Fu F., Chen G. (2014). Graphite-like Carbon Nitrides as Peroxidase Mimetics and Their Applications to Glucose Detection. Biosens. Bioelectron..

[cit75] Basiri S., Mehdinia A., Jabbari A. (2018). A Sensitive Triple Colorimetric Sensor Based on Plasmonic Response Quenching of Green Synthesized Silver Nanoparticles for Determination of Fe 2+, Hydrogen Peroxide, and Glucose. Colloids Surf. A: Physicochem. Eng. Asp..

[cit76] Xi X., Peng X., Xiong C., Shi D., Zhu J., Wen W., Zhang X., Wang S. (2020). Iron Doped Graphitic Carbon Nitride with Peroxidase like Activity for Colorimetric Detection of Sarcosine and Hydrogen Peroxide. Microchim. Acta.

[cit77] Lian J., He Y., Li N., Liu P., Liu Z., Liu Q. (2021). Magnetic Flower-like Fe-Doped CoO Nanocomposites with Dual Enzyme-like Activities for Facile and Sensitive Determination of H_2_O_2_ and Dopamine. Inorg. Chem..

[cit78] Song C., Ding W., Zhao W., Liu H., Wang J., Yao Y., Yao C. (2020). High Peroxidase-like Activity Realized by Facile Synthesis of FeS2 Nanoparticles for Sensitive Colorimetric Detection of H_2_O_2_ and Glutathione. Biosens. Bioelectron..

[cit79] Yuan C., Qin X., Xu Y., Jing Q., Shi R., Wang Y. (2020). High Sensitivity Detection of H_2_O_2_ and Glucose Based on Carbon Quantum Dots-Catalyzed 3, 3′, 5, 5′-Tetramethylbenzidine Oxidation. Microchem. J..

[cit80] Wu K., Li W., Zhao S., Chen W., Zhu X., Cui G., Liu Z., Liu Q., Zhang X., Zhang X. (2020). Cobalt Tuned Copper Sulfide on Montmorillonite: Peroxidase-like Activity, Catalytic Mechanism and Colorimetric Sensing of Hydrogen Peroxide. Colloids Surf. A: Physicochem. Eng. Asp..

[cit81] Yang W., Weng C., Li X., He H., Fei J., Xu W., Yan X., Zhu W., Zhang H., Zhou X. (2021). A Sensitive Colorimetric Sensor Based on One-Pot Preparation of h-Fe3O4@ppy with High Peroxidase-like Activity for Determination of Glutathione and H_2_O_2_. Sens. Actuators, B.

[cit82] Liu Q., Sun Q., Gao W., Shen J., Zhang Y., Lu G., Chen Y., Yu S., Li X. (2022). Turning Built-in Electric Field of Porphyrin on Ti3+ Self-Doped Blue-TiO2 Hollow Nanospheres Boosts Peroxidase-like Activity for High-Performance Biosensing. Chem. Eng. J..

[cit83] McNerney M. P., Zhang Y., Steppe P., Silverman A. D., Jewett M. C., Styczynski M. P. (2019). Point-of-Care Biomarker Quantification Enabled by Sample-Specific Calibration. Sci. Adv..

[cit84] Lister A. S. (2005). 7 - Validation of HPLC Methods in Pharmaceutical Analysis. Sep. Sci. Technol..

