# Supplementary material for: Copper nanocubes as low-cost enzyme mimics in a sarcosine-sensing reaction cascade
Source: Analyst. 2025 Feb 11;150(7):1248–60. doi: 10.1039/d4an01242a (PMC11869937; doi:10.1039/d4an01242a)
Supplement: AN-150-D4AN01242A-s002 [file AN-150-D4AN01242A-s002.pdf]

Supplementary Information (SI) for Analyst.
This journal is © The Royal Society of Chemistry 2025

Supporting information

Copper nanocubes as low-cost enzyme mimics in a sarcosine-sensing reaction cascade

Anuja Tripathi*, Mark P. Styczynski*

School of Chemical and Biomolecular engineering, Georgia Institute of Technology, 950 Atlantic Dr, Atlanta, Georgia 30332, United States

*Corresponding authors email: atripathi84@gatech.edu , mark.styczynski@chbe.gatech.edu

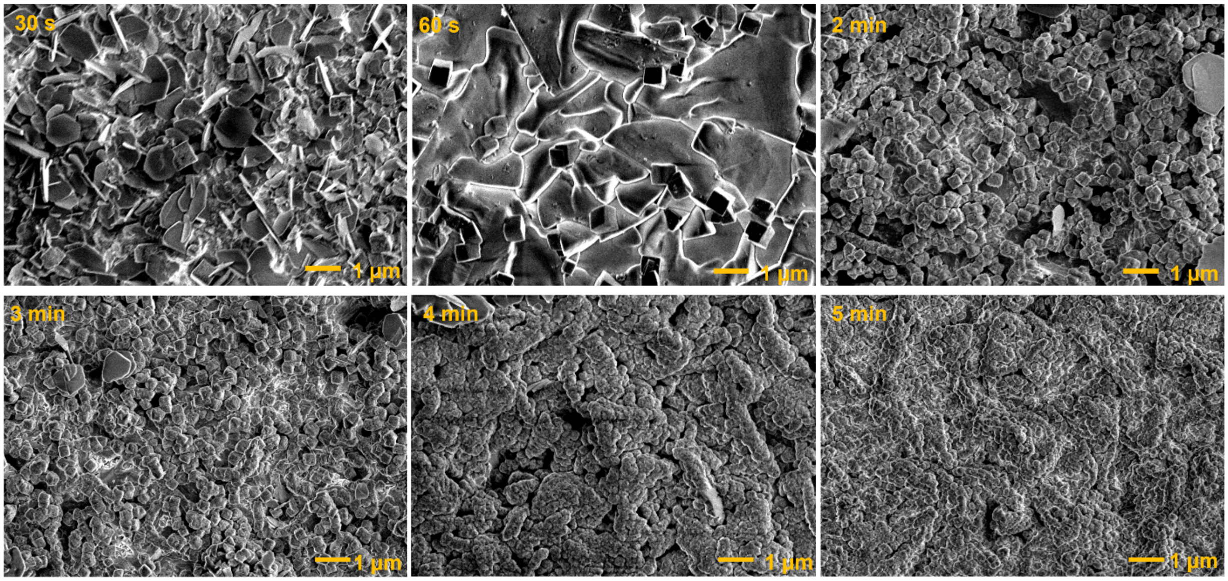

Figure S1: SEM images of etched Cu foil at different time intervals. All samples were etched at constant 8 volts and 4 amperes.

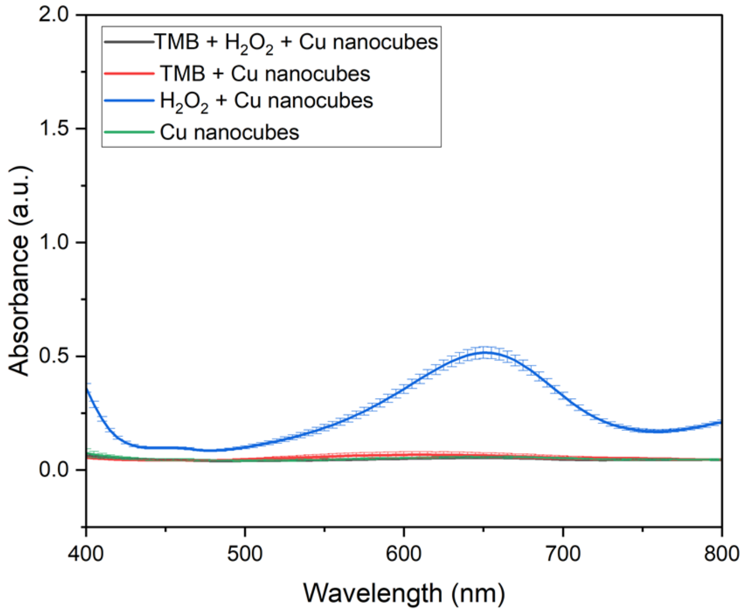

Figure S2: Comparison of absorbance spectra for an aqueous solution containing 1 mM TMB and 100 μM H_2O_2 showing catalysis by Cu nanocubes (blue curve), alongside spectra for solutions containing Cu nanocubes with either 1 mM TMB (red curve) or 100 μM H_2O_2 (black curve), and Cu nanocubes with PBS solution (green curve). Absorbance measurements were taken 30 minutes after immersion of Cu nanocubes in the solution. Error bars represent the standard deviation of three technical replicates.

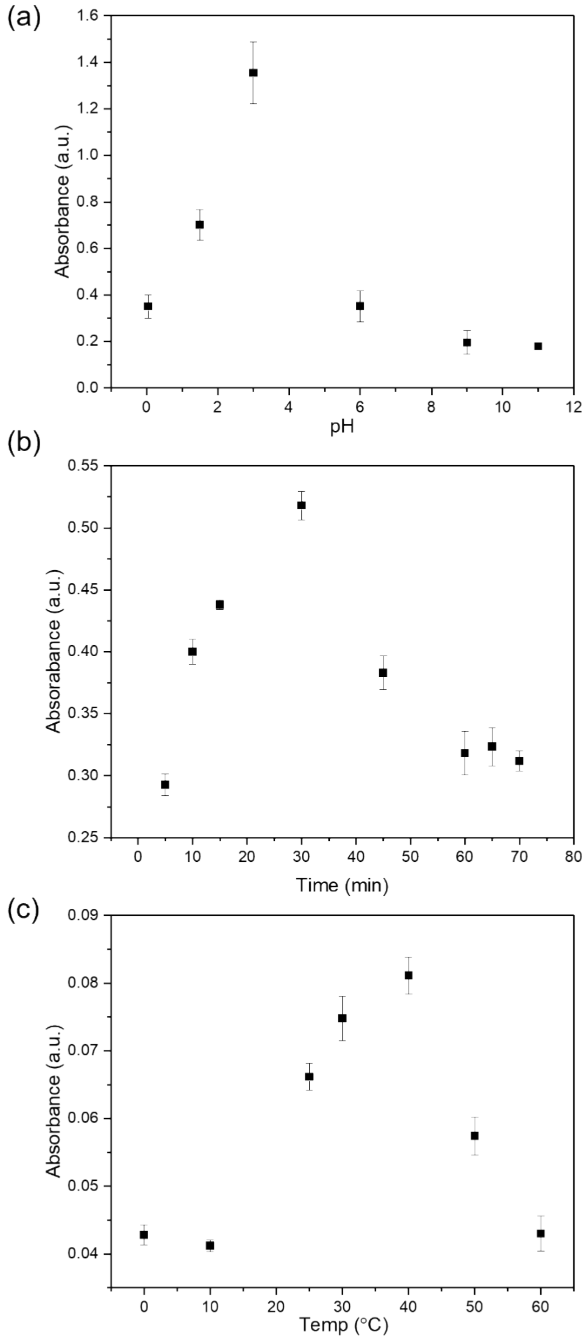

Figure S3: Representative UV-Vis absorbance measurements at 652 nm for optimization of TMB oxidation by Cu nanocubes. (a) Reaction pH was varied at fixed 1mM TMB and 60 μM H_2O_2 concentrations to characterize the pH dependence. (b) Reaction time was varied at fixed 1mM TMB and 60 μM H_2O_2 concentrations to characterize reaction time dependence. (c) Temperature was varied at fixed 1mM TMB and 2 μM H_2O_2 concentrations. Absorbance was recorded after 30 minutes at room temperature. Error bars represent standard deviations of technical triplicate reactions.

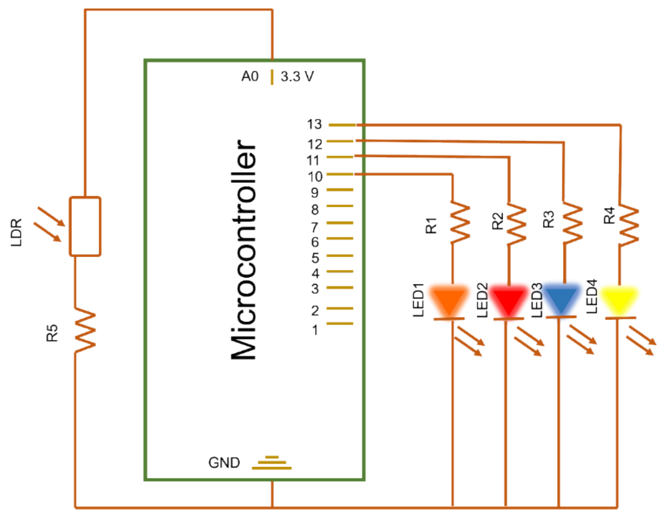

Figure S4: Proof of principle prototype device for visual readout. A schematic circuit diagram for our prototype microcontroller system integrating a light-dependent resistor (LDR), light-emitting diodes (LEDs), and five 10k-ohm resistors.
